# Current and Future Biomarkers in the Diagnosis of Autoimmune Encephalitis: A Review of Biomarker Detection Techniques and Their Performance

**DOI:** 10.3390/medicina62050896

**Published:** 2026-05-06

**Authors:** Patricija Plačenytė, Nataša Giedraitienė, Mantas Vaišvilas

**Affiliations:** 1 Faculty of Medicine, Vilnius University, LT-03101 Vilnius, Lithuania; 2 Clinic of Neurology and Neurosurgery, Faculty of Medicine, Institute of Clinical Medicine, Vilnius University, LT-03101 Vilnius, Lithuania; natasa.giedraitiene@santa.lt (N.G.); mantas.vaisvilas@santa.lt (M.V.)

**Keywords:** autoimmune encephalitis, neuroimmunology, seronegative autoimmune encephalitis, neuronal autoantibodies

## Abstract

Autoimmune encephalitis is an increasingly recognized cause of encephalitis. Detection of disease-specific antibodies is the cornerstone of diagnosis. Despite growing clinical awareness and the routine availability of antibody assays in most centers, diagnosis remains challenging due to diverse clinical presentations, the low diagnostic yield of commercial antibody kits, difficulties in interpreting antibody results, and a substantial proportion of paraclinically silent patients. This narrative review summarizes current diagnostic approaches to autoimmune encephalitis, with particular emphasis on antibody detection strategies, the diagnostic yield of different techniques, serum vs. cerebrospinal fluid testing, and the diagnostic value of supportive cerebrospinal fluid biomarkers. In addition, we discuss patients with seronegative or paraclinically silent disease, in whom diagnosis relies primarily on clinical criteria and the exclusion of alternative etiologies. Finally, we outline future perspectives, including advanced immunological techniques and machine learning-based diagnostic models, which may facilitate earlier identification and more accurate classification of autoimmune encephalitis. Improved integration of clinical assessment with cerebrospinal fluid biomarkers and novel analytical tools may reduce diagnostic delay and support the timely initiation of immunotherapy, ultimately improving neurological outcomes.

## 1. Introduction

In encephalitis of unknown origin, autoimmune encephalitis (AE) has become an increasingly recognized etiology [1]. Traditionally, herpes simplex virus type 1 encephalitis has been considered the most common form of non-endemic encephalitis [2]. However, recent advances in neuroimmunology have identified distinct AE syndromes and novel neural autoantibody biomarkers, fundamentally reshaping the diagnostic approach to these disorders. AE is now understood to encompass both limbic and extra-limbic dysfunction mediated by antibodies against synaptic and intracellular targets. Among the most commonly described antibodies are those targeting N-methyl-D-aspartate receptors (NMDARs), leucine-rich glioma-inactivated protein 1 (LGI1), glutamic acid decarboxylase (65-kD isoform; GAD65), and atypical antibodies or seronegative disease [3]. Despite these advances, diagnosing AE remains challenging due to the broad clinical spectrum, limitations of antibody testing, and often nonspecific findings on conventional paraclinical investigations, including brain magnetic resonance imaging (MRI), electroencephalography (EEG), and routine cerebrospinal fluid (CSF) analysis. This review summarizes current diagnostic strategies for AE, with emphasis on the role of neuronal autoantibody testing in serum and CSF, including available detection methods, their diagnostic performance, and limitations in routine practice. In addition, we discuss potential novel CSF biomarkers to aid in the diagnosis of AE.

## 2. Materials and Methods

To identify relevant literature for this narrative review, a comprehensive search was conducted in the PubMed/MEDLINE database from October 2025 to March 2026. Rather than using predefined automated search strings, literature was identified through manual keyword queries related to the primary study topics, including “autoimmune encephalitis”, “neuronal antibodies”, and “neuroimmunology”, among others. The search strategy was iterative, with additional keywords incorporated as relevant concepts emerged during the review process. To retrieve more specific publications, combinations of relevant keywords were used with Boolean operators, for example: “autoimmune encephalitis” AND “neuronal antibodies”, “autoimmune encephalitis” AND “antibody testing”, and “autoimmune encephalitis” AND “artificial intelligence”. The selection process involved an initial manual screening of titles and abstracts to assess their relevance to the review’s core themes, followed by a detailed evaluation of the full texts of the selected articles. To ensure a comprehensive overview and minimize the risk of selective citation, additional relevant studies were identified through manual cross-referencing of the bibliographies of the selected literature.

Following this selection process, a total of 76 peer-reviewed sources were included in this review. The included literature spans from 2000 to 2026, with a significant emphasis on studies published within the last five years to reflect the most recent advancements in biomarker detection and diagnostic technologies.

Peer-reviewed clinical trials, multicenter prospective or retrospective cohort studies, systematic reviews, meta-analyses, and international expert consensus statements and/or position papers were included in this narrative review.

In contrast, certain types of literature were excluded to maintain the focus on robust clinical diagnostics. Exclusion criteria included single-case reports and small case series unless they reported highly novel and pivotal findings on rare biomarkers or atypical presentations. Furthermore, studies that exclusively focused on the pediatric population and lacked direct clinical translation to adult diagnostics were omitted. Conference abstracts, editorials, and non-English publications were also excluded from the final analysis to ensure that the review is based on fully detailed and peer-reviewed clinical evidence.

## 3. Current Diagnostic Standards

We begin the review by summarizing the established clinical and laboratory standards for diagnosing AE.

### 3.1. Autoimmune Encephalitis Criteria

The first guidelines to aid the diagnosis of autoimmune encephalitis were published in 2016 by Francesc Graus, proposing criteria for the most common forms of AE, namely limbic encephalitis and NMDA receptor encephalitis (NMDARE), using available paraclinical parameters without relying solely on antibody status [4]. These criteria were designed to facilitate early diagnosis and prompt initiation of immunotherapy, given that antibody testing may be delayed or not specific [5].

In clinical practice, the 2016 criteria have been shown to be highly specific for AE when supportive features are present, although a significant proportion of patients with autoimmune encephalopathy may not meet the “possible” criteria due to atypical presentations such as isolated seizures or brainstem involvement [6]. Likewise, some patients with antibodies against VGCC (LGI-1/CASPR2) may progress chronically, have subtle seizures that may be overlooked, or present with dementia syndromes, and therefore not meet the existing criteria [7,8]. Thus, proposed criteria for LGI-1 encephalitis may improve diagnostic yield for some patients [9]. Alternatively, clinical scoring systems developed to predict certain forms of autoimmune encephalitis have been validated externally with good performance metrics [10,11]. Regardless, careful clinical evaluation and reasonable exclusion of mimics remain essential, especially in seronegative patients [6]. The most common conditions that mimic autoimmune encephalitis, which vary across studies, include neurodegenerative disorders, primary psychiatric disorders, functional neurological disorders, cryptogenic epilepsy, brain tumors, neuroinfections, and other rare conditions [5,9,12].

### 3.2. Autoantibody Detection

The detection of autoantibodies in the patient’s serum and/or cerebrospinal fluid is the cornerstone of the diagnosis of definite AE. However, which samples to test and how to test them remain the two most important questions, as testing a sample with a high pre-test probability using the appropriate antibody detection method yields the highest positive predictive value. For example, in NMDARE, the presence of antibodies against NMDAR must be demonstrated in the CSF to minimize false-positive results [13].

The “What” was discussed in the previous section. Briefly, autoimmune encephalitides are relatively well-characterized clinical syndromes that warrant testing when clinical suspicion is high. Cases in which isolated psychiatric symptoms or a single seizure would lead to a diagnosis of AE appear to be uncommon, although such presentations have been described. For example, CSF NMDA antibodies have been reported in patients with isolated primary psychiatric disorders who maintained good symptom control without immunotherapy [13]. Therefore, phenotype-guided antibody detection is likely to improve the positive predictive value, not vice versa.

The “How” is as important as good patient selection. Many centers rely solely on commercial assays for neural antibody detection. Single-tier testing using cell-based assays (CBAs) or line blots is often performed without confirmatory autoantibody detection methods. This approach is justified by its time- and cost-effectiveness, as it allows testing for multiple antibodies with a single assay. However, numerous studies assessing the diagnostic performance of this approach have demonstrated very low sensitivity for many antibodies in serum samples [14]. Specificity tends to be high, partly due to the low incidence of AE and PNS.

**Figure 1:**
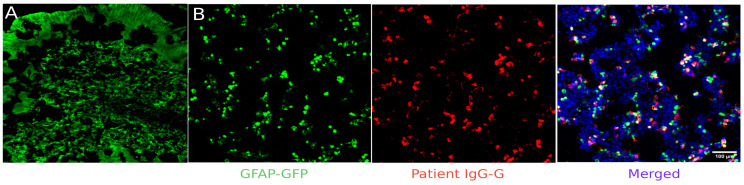
Examples of in-house antigen detection techniques. Demonstration of Glial Fibrillary Acidic Protein (GFAP) on rat brain immunofluorescence and cell-based assays. (**A**) Magnification of rat cerebellum shows intense immunolabeling of astrocytes in the granular layer of the cerebellum, consistent with GFAP. (**B**) GFAP antigen confirmed using HEK293 CBAs overexpressing GFAPα protein (GFAP-GFP); GFAP antibodies in patients’ CSF visualized using secondary anti-human IgG AF647 (Anti-GFAP); Colocalization of GFAP-GFP and anti-GFAP; Images produced in Life Sciences Center, Vilnius University.

An alternative diagnostic approach employed by most academic centers is to use in-house murine brain immunohistochemistry (IHC) as a screening test, followed by targeted antigen-specific testing of positive samples, utilizing mainly CBAs, based on the IHC pattern. This approach may be more cost-efficient because around 95% of samples are negative or show atypical antibodies on initial screening and may not require further testing. Additional advantages of in-house IHC testing include visualization of the entire cerebrum, including the hippocampus and brainstem, which may be important for visualizing several antigens [15,16]. However, the preparation and interpretation of in-house IHC samples require qualified personnel and expertise and are time-consuming. Alternatively, the use of commercially available IHC assays has been reported to be more cost-effective than single CBAs alone [17] and to show comparable performance to in-house IHC in some settings [18], but performance metrics differ between cell-surface and intracellular antibodies [19,20]. Naturally, CSF testing substantially improves diagnostic yield [18,19]. Importantly, utilizing either commercial or in-house IHC assays allows detection of additional antigens that are currently undetectable with commercial CBAs [21]. Examples of antigen detection techniques with in-house assays are shown in Figure 1. Additional research assays include a live hippocampal neuron assay that may be used to demonstrate the surface localization of antibodies of interest [22], but is not the main focus of this review and will not be discussed further.

**Figure 2:**
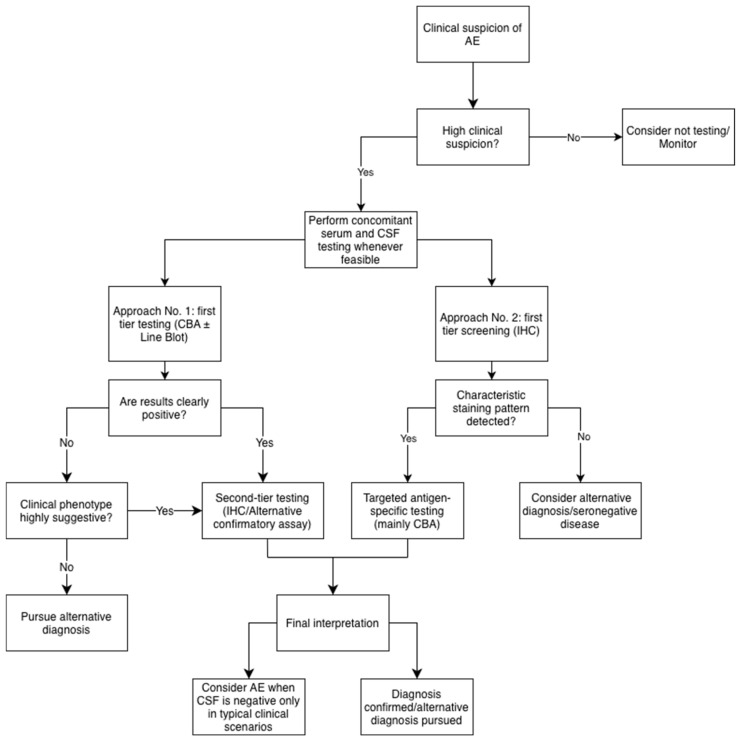
Proposed diagnostic algorithm for neural antibody testing in suspected AE. AE = autoimmune encephalitis; CBA = cell-based assay; CSF = cerebrospinal fluid; IHC = immunohistochemistry.

A proposed diagnostic algorithm for neural antibody detection is shown in Figure 2. Multiple studies have directly compared the two diagnostic strategies shown in Figure 2. Compared with CBAs alone for detecting neural antigens, regardless of whether serum or CSF testing was used, the addition of murine brain immunohistochemistry improved diagnostic yield in academic settings [23]. Likewise, commercial murine tissue-based assays (TBAs) have been shown to be superior to CBAs alone, improving both sensitivity and specificity for detecting neural antibodies [24], identifying additional antibodies [25], and offering good cost-effectiveness compared to CBAs as first-tier testing [17]. To maximize the effectiveness of TBAs, a certain level of expertise is necessary, as interpretation may be challenging. Even with experienced raters, 1/10 samples may be incorrectly classified as positive or negative on TBA. Naturally, the proportion of discordant samples may be higher with inexperienced raters and with serum samples [20]. Additionally, depending on the manufacturer, a significant number of samples show false-negative results with TBA [19].

In contrast, commercial CBA kits, as previously explained, offer the significant advantage of testing for multiple antigens in a single test. However, 1 in 5 samples may yield false-negative results in both serum and CSF, primarily for LGI-1 antibodies [26]. Measures to minimize false-negative results are discussed separately in Section 3.5.

### 3.3. Performance Metrics of Antibody Detection Kits

The sensitivity of individual assays for detecting neural antibodies is reported to be high, likely because negative samples are rarely retested. The specificity of these assays is also very high because AE is very rare; therefore, most negative samples are true negatives [14].

However, the positive predictive value (PPV) of positive serum antibody results can be as low as 5%. This means that, for some antibodies, only 1/20 of positive neural antibody test results may be diagnostic. This applies to most onconeural antibodies when serum is tested without confirmatory assays [27]. Detection of cerebellar degeneration-related protein 2 (CDR2/Yo) by commercial immunodot has the lowest PPV (~5%), whereas antineuronal nuclear antibody 1 (ANNA-1/Hu) positivity has the highest PPV in serum. There appears to be minimal variation in PPV across manufacturers of commercial immunodots [27].

Alternatively, detecting GAD65 antibodies in patients’ serum is the primary cause of incorrect diagnoses of autoimmune encephalitis in adults [12]. Low-titer positivity is associated with misdiagnosis and may be found in healthy individuals [28]. In contrast, high serum and CSF GAD65 titers are associated with classic GAD65 neurological syndromes [29]. Cutoff values for GAD65-associated neurological disorders are consistent across studies and are >10,000 IU/mL in serum and >100 IU/mL in CSF when measured by ELISA [29,30]. An equivalent cutoff for GAD65-associated neurological disorders using radioimmunoassay (RIA) is >20.0 nmol/L in serum and >0.02 nmol/L in CSF; however, the specificity of RIA is lower than that of ELISA [30].

In real-world settings, the inherent limitations of neural antibody detection kits may be mitigated in several ways.

First, screening patient sera with commercial immunodots should be limited to patients with clear, high-risk phenotypes associated with paraneoplastic neurological syndromes. Screening low-risk patients may yield false-positive results [31,32].

Second, using a commercially available tissue-based confirmatory assay should substantially improve diagnostic yield [31,32].

Third, validating positive serum results in CSF will greatly improve diagnostic yield [14].

Finally, in-house laboratory techniques can enhance diagnostic accuracy. For instance, an in-house CDR2L (Yo) CBA assay has demonstrated greater sensitivity than the western blot method in several studies [33,34]. Additionally, detection of other onconeural antigens may be significantly improved by using antigen-specific CBAs, particularly in cases of high clinical suspicion when initial testing is negative [35,36].

### 3.4. Testing Serum Versus Cerebrospinal Fluid

The notion that neural antibodies are more frequently detected in serum than in CSF is not consistently supported and appears to be influenced by the inherent limitations of commercial assays [26]. In many cohorts, approximately 4/5 of patients with AE have disease-specific antibodies in both serum and CSF. Testing serum for neural antibodies without CSF confirmation may be acceptable in cases with highly characteristic clinical presentations; however, because AE can mimic numerous conditions, including primary psychiatric disorders or cryptogenic epilepsy without additional neurological involvement, screening the patient’s serum without CSF confirmation may lead to misdiagnosis of miscellaneous disorders as AE and to the administration of unnecessary immunotherapies, compromising patient safety. For example, around 10% of patients with primary psychiatric disorders harbor serum NMDA antibodies [37]. Likewise, a comparable proportion of serum samples tested positive for various neural antibodies in both primary psychiatric disorders and healthy controls [38]. Ultimately, isolated serum positivity for some antibodies may have little to no positive predictive value, casting doubt on their role in selecting patients for further testing [14].

However, in certain scenarios, serum antibody testing is sufficient, including Myelin Oligodendrocyte Associated Disease (MOGAD) and Neuromyelitis Optica Spectrum Disorder, for which serum testing is generally regarded as the gold standard. However, a small number of patients with rare MOGAD phenotypes may harbor isolated CSF MOG antibodies [39]. Although the significance of this finding is unclear, testing both serum and CSF and assessing concordance between test results should be considered in most cases.

Alternatively, patients with CASPR2 antibody-mediated peripheral nervous system phenotypes, such as Morvan syndrome and Susac syndrome, will typically lack CSF antibodies [40].

Likewise, in non-paraneoplastic Lambert-Eaton myasthenic syndrome (LEMS), detection of voltage-gated calcium channel antibodies in serum is sufficient. However, small cell lung cancer-associated LEMS will show either Hu or SOX-1 positivity [41]. CSF testing and confirmatory assays may be required to confirm Hu and SOX-1 antibody positivity [29].

### 3.5. Navigating Between Underdiagnosis and Overdiagnosis

**Figure 3:**
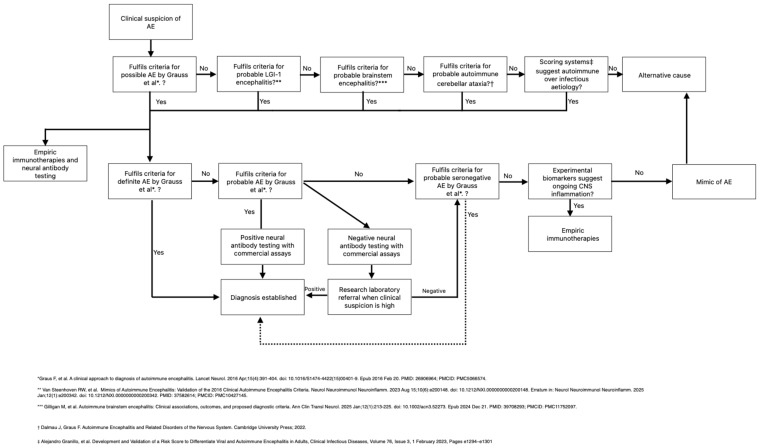
A practical workflow for the diagnosis of AE. Solid arrows indicate the standard diagnostic flow; the dotted arrow indicates that a diagnosis of seronegative AE can be established if the specific Grauss et al. criteria are fulfilled. * [4], ** [9], *** [63], † [64], ‡ [65].

The current criteria for identifying AE and its subsets (Figure 3), along with commercially available neuronal antibody detection kits, increase the likelihood of accurate diagnosis and timely treatment. However, these established criteria may lead to misidentification of a subset of patients with AE who present with atypical clinical symptoms. For example, a small proportion of patients may have an insidious onset and present with dementia without paraclinical evidence of inflammation [7]. Additionally, peripheral neuropathy, movement disorders, or motor neuron disease phenotypes may rarely be encountered in different antibody-mediated neurological syndromes [41,42]. Although atypical, many of these patients may exhibit subtle seizures that are easily overlooked, or they may present with symptoms that do not meet the established criteria for motor neuron diseases or movement disorders. It is essential to maintain a high level of suspicion and a low threshold for neural antibody testing in this population. Another contributor to underdiagnosis may be the absence of supportive paraclinical features that would confirm a diagnosis of AE. These scenarios are discussed separately in Section 5 and Table 1.

At the laboratory level, false-negative antibody results should be managed using several strategies. These include repeat testing, discussions among CBA/TBA evaluators about discordant samples, careful correlation of antibody results with the clinical presentation, and, ultimately, referral to research centers for complex cases. It is important to note that delays in antibody detection should not postpone immunotherapies for patients with suspected AE.

In contrast, a substantial rate of false-positive antibody test results, particularly for onconeural antibodies, may lead to overdiagnosis of AE. To mitigate the risk of overdiagnosis, certain practices should be adopted.

First, although neural antibodies are rarely detected in the serum of healthy controls, they are not uncommon in inflammatory and non-inflammatory neurological disorders [62]. Therefore, as explained in previous sections, testing for onconeural antibodies should be guided by a high clinical suspicion of an underlying autoimmune nervous system disorder to maximize pre-test probability.

Second, to maximize diagnostic accuracy, CSF confirmation should generally be obtained whenever available. However, this does not apply to CAPSR2 antibody-associated peripheral nervous system phenotypes, such as Morvan syndrome and neuromyotonia, which lack detectable CSF antibodies [40].

Third, positive onconeural antibodies should be confirmed by immunohistochemistry. However, immunohistochemical detection of antigens such as Ma2 can be particularly challenging due to nonspecific staining patterns. Therefore, confirmation with CBA may be necessary.

Lastly, quantitative evaluation of certain antibodies may be necessary to confirm the diagnosis (GAD65; see the Section 3.2 for detailed recommendations on GAD65 testing).

## 4. Emerging and Experimental Biomarkers

The diagnostic landscape of autoimmune encephalitis (AE) is evolving, with emerging biomarkers showing varying degrees of clinical readiness and utility. While some may prove diagnostic, most currently play supportive or exploratory roles in the diagnostic process. Below, we summarize potential future biomarkers under investigation for diagnosing or prognosing AE.

### 4.1. Oligoclonal Bands

While the role of oligoclonal bands (OCBs) in AE remains uncertain, they are among the most extensively evaluated ancillary tests to support an autoimmune origin in cases of encephalitis of unknown etiology. Therefore, OCBs may serve as a complementary biomarker in the following scenarios.

In antibody-negative AE, OCBs may indicate an autoimmune etiology in about one-fifth of patients, complementing other supportive MRI and cerebrospinal fluid (CSF) findings [66].

In contrast, in definite GAD65 antibody-mediated neurological disorders, OCB positivity was reported as the sole paraclinical abnormality in two-thirds of patients [29,67]. The positivity rate varies but is usually lower in VGCC antibody-mediated encephalitides and IgLON-5 disease [68]. However, inconsistent testing and variable results across studies make interpreting this test difficult. Ultimately, OCBs can support the diagnostic process, but they are not a reliable standalone test for confirming a diagnosis.

### 4.2. Kappa Free Light Chains

Although some reports suggest that the kappa free light chain (κ-FLC) index may be a more sensitive ancillary tool for demonstrating intrathecal B-cell activation than OCBs [69], the evidence is inconclusive, with other reports showing poor discrimination between primary psychiatric and inflammatory etiologies [70]. Moreover, positive cut-off values specific to the AE population have not been established, and the test may struggle to distinguish AE from infectious encephalitis or multiple sclerosis [68,71]. Ultimately, while it shows promise, this biomarker is not sufficient for diagnosis on its own but may be valuable when used alongside other tools in the right clinical context.

### 4.3. Neurofilament Light Chain Measurements

Serum and CSF neurofilament light chain (NfL) measurements do not appear to have diagnostic value. However, they may provide additional information about disease activity and long-term prognosis in AE, although their direct association with clinical severity, prognosis, or other paraclinical features remains variable across studies [72,73].

### 4.4. CSF Flow Cytometry for Plasma Cell Detection

Identifying CSF-bound plasma cells is increasingly studied as a potential biomarker for antibody-positive CNS autoimmune disorders. However, due to limited data, its role as a diagnostic or supportive marker remains unclear [74].

A diagnostic workflow that incorporates established clinical and laboratory criteria, as well as the clinical application of experimental biomarkers, is shown in Figure 3.

## 5. Diagnosis in Cases with Normal Paraclinical Findings

A considerable proportion of AE syndromes may present with normal ancillary testing despite a definite inflammatory etiology. In many of these cases, the conditions are chronically progressive, associated with specific antibodies, and therefore may have a characteristic clinical presentation. For example, LGI1-antibody–associated AE may present with isolated faciobrachial dystonic seizures (FBDS), which are easily overlooked yet highly characteristic clinical features [43]. Similarly, CASPR2-antibody–associated syndromes may present with limbic encephalitis, Morvan syndrome, or peripheral nerve hyperexcitability, with indolent progression and a risk of misdiagnosis as polyneuropathy of an alternative etiology [75]. Detailed clinical phenotypes, corresponding antibodies, their cancer associations, and other features are shown in Table 1.

## 6. Future Considerations

Despite substantial progress in understanding and diagnosing AE, several important challenges remain. One critical area is refining diagnostic strategies for patients with normal paraclinical findings [4]. Algorithms and updated clinical criteria may be necessary to identify cases that progress silently without abnormalities on ancillary testing.

Seronegative AE highlights another important direction for future research, suggesting the presence of yet unidentified antigenic targets or limitations in current assay sensitivity [48]. Applying machine learning or data-driven models to large datasets might enable the recognition of subtle patterns predictive of autoimmune etiology, even when individual paraclinical modalities are normal, and aid in differentiating between infectious and autoimmune etiologies [76].

Despite encouraging performance, several important limitations of machine learning approaches in autoimmune encephalitis warrant consideration. Many studies rely on relatively small, often single-center datasets, which may compromise robustness, reduce generalizability, and increase the risk of overfitting to patterns specific to the internal cohort [77,78]. Class imbalance and underrepresentation of certain AE subtypes may further degrade predictive performance. Practical implementation limitations, including the need for specialized imaging, standardized acquisition protocols, and complex data analysis workflows, may restrict applicability in routine clinical practice, particularly in resource-limited settings [78]. In addition, heterogeneity in imaging data, reliance on manual segmentation, and variability in laboratory workflows may introduce bias and reduce reproducibility across centers [77]. Overall, larger multicenter datasets, standardized methodologies, and prospective external validation are needed to improve generalizability and support clinical translation of these approaches.

## 7. Conclusions

AE represents a complex and heterogeneous group of disorders in which clinical judgment and demonstration of neuroinflammation using paraclinical parameters remain central to diagnosis. Normal MRI, EEG, and routine CSF findings do not exclude the diagnosis, whereas positive antibody results should be carefully correlated with the clinical synopsis to avoid misdiagnosis and unnecessary immunotherapies. A multi-step antibody detection approach may be necessary to ensure diagnostic accuracy. While antibody detection in the CSF is still widely considered the reference standard, oligoclonal bands, intrathecal free kappa light chains, and emerging cytokine or chemokine profiles may also support the diagnosis. Continued research into seronegative and paraclinically silent presentations will likely be essential to further optimize patient outcomes and expand our understanding of the immunopathology underlying these disorders.

## Figures and Tables

**Table 1:** Clinical features, disease course, and tumor prevalence in selected AE-associated antibodies [8,41,42,43,44,45,46,47,48,49,50,51,52,53,54,55,56,57,58,59,60,61].

Antibody	Phenotype/Clinical Features	Typical Course	Tumor Association/Prevalence	Proportion of Cases with Normal Ancillary Tests
LGI-1	Limbic encephalitis, cognitive impairment, seizures, neuropsychiatric disorders, hyponatremia [43] May present with subtle isolated FBDS	Mostly chronic, high risk of relapse (~28%) [8]	Tumor association weak (<10%) [44]	CSF negative for OCB’s and pleocytosis in 89% of cases [45] MRI normal in 72% of cases [45]
CASPR2	Limbic encephalitis, Morvan syndrome, peripheral nerve hyperexcitability (Isac syndrome); Paroxysmal and kinetic movement disorders: ataxia, orthostatic paroxysmal myoclonus and continuous spinal myoclonus [46]	Mostly chronic symptom progression; symptom nadir >12 months in peripheral syndromes [47]; elevated risk (~25%) of relapse	Tumor association uncommon (~20%); mostly thymomas or small cell lung cancers [48]	CSF negative for OCB’s and pleocytosis in 60–70% of cases [46] MRI negative ~60% of cases [46]
IgLON-5	Sleep disorders (parasomnia), bulbar dysfunction, cognitive impairment, PSP-like phenotypes, movement disorders [49]	Chronic/progressive in most cases. Median time to diagnosis from symptom onset ~4 years [50]	No consistent tumor link reported [48]	CSF negative for OCBs in 90% of cases. Normal cellularity in 80% [51,52] MRI normal or regional brain atrophy patterns, dependent on the dominant disease phenotype [50]
GAD65	Limbic encephalitis, Stiff-Person Syndrome, cerebellar ataxia, temporal lobe epilepsy; Small fraction of patients may resemble motor neuron disease [42]	Chronic onset with slow progression [53]	Tumor association very weak (~5%); mostly thymomas and less often breast, thyroid, renal, colon cancers [53]	CSF normal cellularity in 90%, OCB’s may be present in ~40–70% of cases [54,55] MRI negative or cerebellar atrophy in 100% with cerebellar ataxia [55] Hippocampal atrophy in ~50%, less frequently inflammatory or post-ictal hippocampal lesions [56]
Ri	Cerebellar syndrome, isolated tremor (or other movement disorders), oculomotor disturbances, PSP-like phenotypes, atypical parkinsonian features, opsoclonus-myoclonus syndrome [57]	Multistep disease course, often progressive [57]	Tumor association very strong (~90%); most commonly breast cancer [57]	Normal CSF cellularity in ~70% [57] OCBs frequent (~80%) [57] MRI normal in 80% of cases [57]
Ma2	Limbic, diencephalic, brainstem encephalitis, narcolepsy-cataplexy, movement disorders, peripheral neuropathy, motor neuron disease type presentations, isolated brain sarcoidosis [41,58,59,60,61]	Chronic disease course in 70% [41]	Tumor association strong (~75%); Many cases with testicular or lung malignancies [41]	Normal CSF in ~50% [41] MRI negative in ~30% [41] Hippocampal or cerebellar atrophy may be present [41]

## Data Availability

No new data were created or analyzed in this study.

## Abbreviations

The following abbreviations are used in this manuscript:

AE	Autoimmune encephalitis
NMDAR	N-methyl-D-aspartate receptors
LGI1	Leucine rich glioma-inactivated protein 1
GAD65	Glutamic acid decarboxylase
MRI	Magnetic resonance imaging
EEG	Electroencephalography
CSF	Cerebrospinal fluid
VGCC	Voltage-gated calcium channel
CBA	Cell-based assay
PNS	Paraneoplastic neurological syndrome
IHC	Immunohistochemistry
GFAP	Glial fibrillary acidic protein
Caspr2	Contactin-associated protein-like 2
GABABR	Gamma-aminobutyric acid type B receptor
IgLON5	Immunoglobulin-like cell adhesion molecule 5
AMPAR	α-amino-3-hydroxy-5-methyl-4-isoxazolepropionic acid receptor
MOGAD	Myelin oligodendrocyte associated disease
OCB	Oligoclonal band
κ-FLC	Kappa free light chain
NfL	Neurofilament light chain
FBDS	Faciobrachial dystonic seizures
PSP	Progressive supranuclear palsy
RIA	Radio-immuno assay
